# Prevalence and Pathogenic Factors of Thyroid Dysfunction in First-Episode and Drug-Naïve Major Depressive Disorder Patients With Fasting Blood Glucose Abnormalities in Early- and Late-Onset Age

**DOI:** 10.1155/da/9947375

**Published:** 2025-02-28

**Authors:** Ting Wang, Minxuan Zhang, Jinjin Cao, Sanrong Xiao, Xiangyang Zhang

**Affiliations:** ^1^Department of Humanities, Jiangxi University of Chinese Medicine, Nanchang, Jiangxi, China; ^2^School of Public Policy and Administration, Nanchang University, Nanchang, Jiangxi, China; ^3^Hefei Fourth People's Hospital, Hefei, Anhui, China; ^4^Affiliated Mental Health Center of Anhui Medical University, Hefei, Anhui, China; ^5^Anhui Mental Health Center, Hefei, Anhui, China

**Keywords:** abnormal fasting blood glucose, major depressive disorder, thyroid dysfunction

## Abstract

**Aims:** This study aims to explore the mutual mechanisms and distinct pathogenic factors between fasting blood glucose (FBG) abnormalities and thyroid dysfunction (TD) in major depressive disorder (MDD) patients of different onset ages.

**Methods:** One thousand seven hundred eighteen first-episode and drug-naïve (FEDN) MDD patients were selected. Hamilton Depression Rating Scale (HAMD), Hamilton Anxiety Rating Scale (HAMA), Positive and Negative Syndrome Scale (PANSS) positive subscale, Clinical Global Impression (CGI), FBG, and thyroid-stimulating hormone (TSH) were measured, along with other relevant biochemical indicators.

**Results:** TD prevalence was 86.69% in early-onset MDD patients with abnormal FBG while in late-onset was 86.86%. No significant difference was found. The area under the curve (AUC) values of FBG detecting TD were all over 0.700. Depressive symptoms and lipid metabolites were significant risk factors and were more specific indicators for late-onset MDD patients with FBG abnormalities. Further binary logistic regression and receiver operating characteristic (ROC) curves revealed that depression severity, high-density lipoprotein cholesterol (HDL-C) predicted TD well in MDD patients with FBG abnormalities, making this predictive effect more significant in the late-onset group.

**Conclusions:** Insulin resistance and lipid metabolism abnormalities based on FBG abnormalities significantly impact TD in late-onset MDD. Specificity and regular monitoring should be considered for different onset ages of MDD patients with abnormal metabolism. Further research should clarify the interactions among insulin resistance, lipid metabolism, and TD. The First Hospital of Shanxi Medical University Ethics Committee reviewed and approved this study (No. 2016-Y27).

## 1. Introduction

Major depressive disorder (MDD) is characterized by chronic reduced emotion, decreased interest, and cognitive impairment, frequently occurring during adolescence and adulthood, with varied symptoms. According to an investigation in 2011, 21 million adults had MDD episodes, and 14.5 million adults had MDD episodes with severe impairment in the United States [1]. Data from 2013 to 2015 show that, the lifetime prevalence of depressive disorder was 6.8% and of MDD was 3.4% [2]. Between 2021 and 2022, the detection rate of depression risk in Chinese adults was 10.6% [3]. The number of Chinese depression cas were more than 95 million [4]. Up to 2021, there was an increase in Chinese depression cases of 54% in the past 30 years [5]. MDD is becoming common and co-occurs with multiple other diseases [1]. Psychiatric disorders, including MDD, are often associated with metabolic illnesses, especially in midlife or older age population [6–9]. Several metabolic pathophysiological processes have been identified in MDD patients. MDD patients exhibited disturbances in glycine and serine metabolism [10], leading to varying degrees of neurological symptoms as well as metabolic disorders. Hatch found that MDD subjects with metabolic disorders exhibited more severe deficits in the whole brain, white matter, and subcortical volume than those without metabolic illnesses, independent of antidepressant medication [8]. Onset age differences correlate with the differences in MDD symptoms and influence metabolic diseases. There was a negative correlation between subgenual anterior cingulate cortex and hippocampal volumes and the severity of depression, and this relationship was found to be more statistically significant in adult MDD episodes [11]. Furthermore, adults with early-onset MDD were more likely to be accompanied by suicidal attempts compared to those under 30 years old [12]. Late-onset MDD patients (>50 years) experienced more significant weight loss and more severe gastrointestinal symptoms than those aged less than 30 years [13]. An enhanced understanding of the impact of onset ages on MDD will improve clinical assessment and care, especially in comorbid MDD cases.

MDD is also accompanied by mild neuroendocrine disorders, such as thyroid dysfunction (TD). TD is a joint group of congenital or acquired diseases characterized by increased or decreased secretion of thyroid hormones, including overt and subclinical hyperthyroidism, overt and subclinical hypothyroidism, autoimmune thyroiditis, and thyroid cancer. Both the hypothalamic–pituitary axes change due to depressive symptoms, and the dopamine in the monoamine hypothesis of depression indicated a close correlation between these two diseases [14, 15]. Additionally, TD patients were more susceptible to developing depression, which remained most common in hyperthyroidism and hypothyroid patients [16]. According to work by Krausz et al. [17], different neural circuits in hypothyroidism may mediate behavioral symptoms in depression. Fasting blood glucose (FBG) is a crucial indicator of glucose metabolism abnormalities. Diabetes is a more prevalent illness among MDD patients compared to healthy populations and is associated with shortened lifespans [3, 14, 18]. Gender differences in lipid markers existed among MDD patients with abnormal FBG [19]. A case-controlled study demonstrated significantly reduced insulin sensitivity and impaired glucose tolerance in MDD patients [20].

Clinical practices also highlight the complex interplay between glucose metabolism and TD [21]. Compared to non-suicide attempters, Liu et al. [9] observed statistically higher serum thyroid-stimulating hormone (TSH), glucose, antithyroglobulin (A-TG), antithyroid peroxidase (A-TPO) in MDD patients with suicidal attempts had. At present, research mainly focused on the relationship between type 2 diabetes and thyroid functions, such as the interactions of the pancreas and the thyroid, insulin and TSH, adenosine monophosphate-activated protein kinase (AMPK), and glucose transporter [22, 23]. Patients with diabetes mellitus may be detected as new cases of TD [24]. Partially pancreatectomized would develop hyperglycemia when treated with thyroid extracts, and diabetes status would be exhibited when treated with thyroid powder for the long term [22]. Patients with diabetes had decreased conversion of T4 into T3, and the pancreatic islet from hypothyroid subjects secreted less [25]. Some researchers paid much attention to the interactions between insulin sensitivity or resistance and thyroid-related secretions [20]. Thyroid hormone replacement therapy (HRT) would regulate the sensitivity of insulin in female hypothyroidism patients [26]. Some other studies were interested in type 1 diabetes and thyroid autoimmunity, and the results were mainly linked to genetic background and susceptibility [22, 27]. However, the interactive mechanisms among MDD, glucose metabolism, and thyroid function require further elucidation.

Research on the effect of TSH hormone on metabolic syndrome in patients has been carried out by Zhang's et al. [28] team; however, the findings indicate that TSH can accurately predict the occurrence of metabolic syndrome and that patients with depression who exhibited abnormal thyroid hormones possess distinct biochemical indicators [29]. In order to get a more thorough explanation of the physiological mechanisms, we still intend to perform a more thorough investigation and concentrate on the impact mechanism of aberrant glucose metabolism on TD in patients with varying ages of MDD onset.

Only a few studies have explored the mechanisms between glucose metabolism and TD in MDD patients, with a minority diagnosed significantly with TD in previous research, and there has been a lack of relevant research in recent years. Most studies focus on gender differences rather than variations in onset age and pathogenic factors. Therefore, this study aims to explore the prevalence of TD based on glucose metabolism abnormalities in MDD patients and elucidate the association with different onset ages of MDD and remarkably diverse pathogenic mechanisms. To the best of our knowledge, no research has examined the correlations between FBG impairment in different onset age groups of MDD patients and the incidences of TD.

## 2. Material and Methods

### 2.1. Subjects

From 2015 to 2017, we selected 1718 participants from the Department of Psychiatry at the First Hospital of Shanxi Medical University in Taiyuan City, Shanxi Province, China. Two hundred thirty-four patients of these participants had abnormal FBG. The main characteristics are shown in Table 1. All patients participated in the study with informed consent. The First Hospital of Shanxi Medical University Ethics Committee reviewed and approved this study (No. 2016-Y27). The data used for this research are secondary analysis, and the author intends to learn more about the effects of the age at which MDD first manifests.

The criteria for participant recruitment were (1) age range from 18 to 65 years, Chinese Han nationality; (2) first-episode and drug-naïve (FEDN) patients who were diagnosed with MDD, according to the DSM-IV; (3) did not use antidepressant, antipsychotic drugs, and any other medications; and (4) diagnosed with MDD only and no any severe physical diseases or organic brain diseases.

### 2.2. Clinical Assessment

We collected sociodemographic information by questionnaires, including gender, age, marital status, and educational background. We used the Hamilton Depression Rating Scale (HAMD)-17 and Hamilton Anxiety Rating Scale (HAMA)-14 to obtain the scores for depression and anxiety, respectively, to evaluate the degree. We also employed the Positive and Negative Syndrome Scale (PANSS) positive subscale to positive psychiatric symptoms. Nurses and doctors in the work were trained to use these scales correctly, and each patient was assessed independently by the two professionally trained researchers mentioned above. Anxiety levels of patients were divided into four types, according to a total score of the HAMA: (1) HAMA score ≤ 7 was considered to be without anxiety; (2) 8 ≤ HAMA score ≤ 14 was maybe anxiety; (3) 15 ≤ HAMA score ≤ 21 has mild anxiety; (4) 22 ≤ HAMA score ≤ 29 had moderate anxiety; and (5) HAMA score ≥ 29 had severe anxiety. Patients whose PANSS positive subscale score exceeded 15 were supposed to be with psychiatric symptoms. In this study, our cutoff value of onset age is 30 years old. Early-onset MDD patients' onset ages were less than 30 (including 30), and late onsetters were exceeding 30 years old [13, 30].

### 2.3. Measurement of Physical and Biochemical Indicators

We collected fasting blood samples of recruited patients between 6 am and 8 am before they received any drugs. We also gathered serum levels of several biochemical parameters: TSH, A-TPO, A-TG, free triiodothyronine (FT3), free thyroxine (FT4), total cholesterol (TC), total triglycerides (TGs), high-density lipoprotein cholesterol (HDL-C), low-density lipoprotein cholesterol (LDL-C), and FBG. Trained nurses collected the clinical information of patients, including blood pressure. In this study, the definition of abnormal FBG and TD is (1) hyperglycemia, FBG ≥ 6.1 mmol/L; (2) hypertension, systolic blood pressure ≥ 140 mmHg and/or diastolic blood pressure ≥ 90 mmHg; (3) abnormal A-TG, A-TG ≥ 115 IU/L; (4) abnormal A-TPO, A-TPO ≥ 34 IU/L; (5) TSH > 4.2 mIU/L and 10 ≤ FT4 ≤ 23 pmol/L were considered to be subclinical hypothyroidism; (6) hyperthyroidism, TSH < 0.27 mIU/L and FT4 exceeding 23 pmol/L; (7) TSH exceeding 4.2 mIU/L and accompanied by FT4 < 10 pmol/L was hypothyroidism; and (8) thyroid autoimmunity, any of abnormal A-TG or abnormal A-TPO.

### 2.4. Statistical Analysis

The Shapiro–Wilk Test was used to confirm whether continuous variables were normal distributions. Continuous variables that conform to normal distribution were tested by analysis of variance (ANOVA), while the Mann tested those that did not conform to normal distribution–Whitney *U* test. The chi-square test was used to analyze categorical variables. To explore TD risk factors of MDD patients with FBG abnormalities, we performed different tests between the early-onset MDD group and the late-onset MDD group. The data of different onset age groups were analyzed by multiple linear regression and binary logistic regression analysis, with the biochemical indicators of thyroid function as dependent variables. All data were performed on SPSS 27.0.1, and the significance level in this study was 0.05.

## 3. Result

### 3.1. Prevalence of TD Among All MDD Participants and the Prediction of FBG for TD

**Figure 1:**
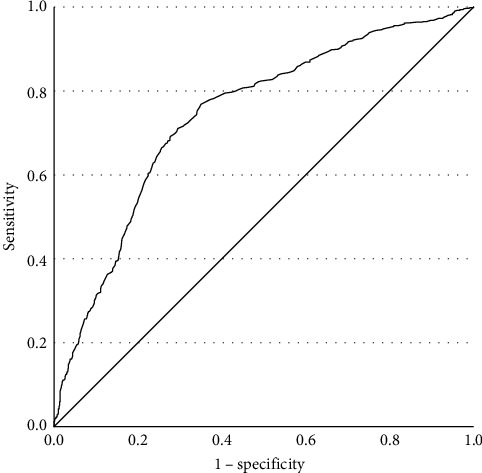
ROC curve of FBG in all MDD patients. FBG, fasting blood glucose; MDD, major depressive disorder; ROC, receiver operating characteristic.

The prevalences of various TDs are shown in Table 2. There were no subjects with overt hyperthyroidism or subclinical hyperthyroidism in this study. Only one patient without FBG abnormalities suffered overt hyperthyroidism. Compared to MDD patients without abnormal FBG, higher TSH levels were observed in patients with FBG abnormalities (*t* = 12.913, *p*  < 0.001). We conducted binary logistic regression analysis and found that FBG had a significant association with TD (*β* = 1.334, *p*  < 0.001, OR = 3.797, 95% CI [3.063, 4.707], Cox and Snell *R*^2^ = 0.278). The receiver operating characteristic (ROC) curve of FBG is shown in Figure 1. The area under the curve (AUC) was 0.722 (95% CI [0.697, 0.748], *p*  < 0.001). The Youden index was 0.386, and the cutoff value was 5.145, with a sensitivity of 0.732 and a specificity of 0.653. The positive predictive value (PPV) was 72.2%, and the negative predictive value (NPV) was 59.7%.

**Figure 2:**
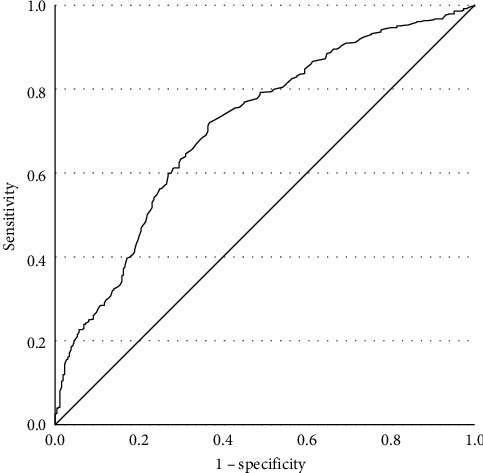
ROC curve of FBG for early-onset group. FBG, fasting blood glucose; ROC, receiver operating characteristic.

Among early-onset MDD patients (aged 18–30 years old, 22.90 ± 4.263), prevalences of TD are shown in Table 3. We conducted a binary logistic regression with TD as the dependent variable, finding that FBG was statistically significant (*β* = 1.160, *p*  < 0.001, OR = 3.191, 95% CI [2.073, 4.913], Cox and Snell *R*^2^ = 0.483), which indicated that early-onset MDD patients with FBG abnormalities had 3.191 times greater risk of developing TD compared to those without abnormal FBG. The ROC curve was plotted (Figure 2). The effect of FBG in predicting TD in the early-onset group was substantial, with an AUC of 0.710 (*p*  < 0.001, 95% CI [0.671, 0.748]). Youden index was 0.352, sensitivity was 0.720, and specificity was 0.633. The cutoff value was 5.145. The PPV was 71.3%, and the NPV was 63.6%.

**Figure 3:**
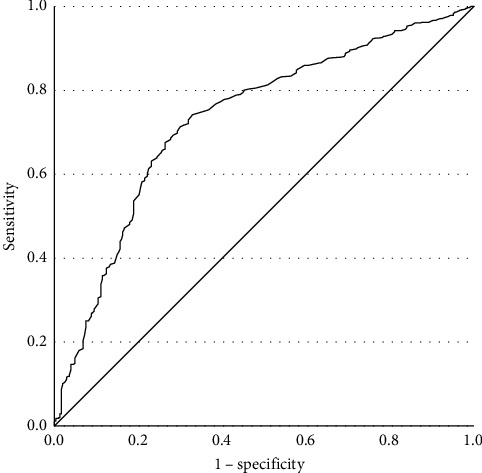
ROC curve of FBG for late-onset group. FBG, fasting blood glucose; ROC, receiver operating characteristic.

 For patients with late-onset MDD (aged 31–60 years old, 44.15 ± 7.755), prevalences of TD are shown in Table 4. A binary logistic regression was employed with TD as the dependent variable. FBG (*β* = 1.200, *p*  < 0.001, OR = 3.320, 95% CI [2.327, 4.739], Cox and Snell *R*^2^ = 0.431) was statistically significant. Late-onset MDD patients with FBG abnormalities had a 3.320 times greater risk of developing TD compared to those without abnormal FBG. The ROC curve is shown in Figure 3 (AUC = 0.732, *p*  < 0.001, 95% CI [0.698, 0.766]). Youden index was 0.413, sensitivity was 0.742, and specificity was 0.671. The cutoff value was 5.145. The PPV was 72.9%, and the NPV was 56.2%.

No differences in prevalences of TD, subclinical hypothyroidism, A-TG abnormalities, A-TPO abnormalities, and thyroid autoimmunity were found between early- and late-onset MDD patients.

### 3.2. Differences Between Different Onset Ages of MDD Patients With FBG Metabolism Abnormalities and Factors

The biochemical indicators of the two onset groups are shown in Table 5. Patients with late-onset MDD had a longer duration of MDD, according to our research. Although there were no statistically significant differences in the severity of depression among age groups, the late-onset group had greater levels of depression. Due to the small number of individuals with hypertension and abnormal BMI in our sample, we regarded these data as control variables.

For early-onset MDD patients with FBG abnormalities, based on the results of relevant analysis, we conducted a multiple linear hierarchical regression analysis and binary logistic regression analysis, using onset ages, gender, education level, marital status, FBG levels, and so on as control variables. The results showed that TC (*β* = 0.682, *p*=0.013, 95% CI [0.147, 1.216], VIF = 2.473) and systolic blood pressure (*β* = 0.087, *p*=0.004, 95% CI [0.029, 0.146], VIF = 1.993) were significantly associated with TSH level (*R*^2^ = 0.493, adjusted *R*^2^ = 0.414, *F* = 6.216, *p*  < 0.001). The course of MDD (month) was significantly related to FT4 level (*β* = −0.301, *p*=0.002, 95% CI [−0.487, −0.116], VIF = 1.111); however, the corrected model was not significant (*F* = 1.603, *p*=0.101), and the *R*^2^ was 0.201, leading to an adjusted *R*^2^ value of 0.076. Moreover, BMI was correlated with abnormal A-TG (*β* = −1.178, *p*=0.014, OR = 0.308, 95% CI [0.121, 0.785], Cox and Snell *R*^2^ = 0.598), and TC significantly predicted abnormal A-TPO (*β* = 0.599, *p*=0.039, OR = 1.820, 95% CI [1.031, 3.213], Cox and Snell *R*^2^ = 0.458).

For patients with late-onset MDD, we employed forward LR method for hierarchical regression to control for variables. PANSS positive subscale scores (*β* = 0.195, *p*  < 0.001, 95% CI [0.137, 0.253], VIF = 1.141), TC (*β* = 0.958, *p*  < 0.001, 95% CI [0.626, 1.289], VIF = 1.310), and HDL-C (*β* = −1.826, *p*=0.002, 95% CI [−2.958, −0.693], VIF = 1.259) were associated with TSH (*R*^2^ = 0.597, adjusted *R*^2^ = 0.554, *F* = 14.004, *p*  < 0.001). LDL-C predicted FT3 significantly (*β* = −0.143, *p*=0.022, 95% CI [−0.265, −0.021], VIF = 1.175); however, the model fit was relatively poor (*R*^2^ = 0.164, adjusted *R*^2^ = 0.090, *F* = 2.222, *p*=0.017). TG was significantly related with FT4 (*β* = −0.543, *p*=0.022, 95% CI [−1.008, −0.079], VIF = 1.093), with an *F* of 2.450 (*p*=0.008), an *R*^2^ of 0.177, and an adjusted *R*^2^ of 0.105. When abnormal A-TPO was the dependent variable in binary logistic regression, TC (*β* = 0.617, *p*=0.007, OR = 1.853, 95% CI [1.186, 2.895], Cox and Snell *R*^2^ = 0.372) exhibited significant correlations. When abnormal A-TG was the dependent variable, HDL-C (*β* = −1.669, *p*=0.049, OR = 0.188, 95% CI [0.036, 0.996], Cox and Snell *R*^2^ = 0.210) was significantly correlated with it.

### 3.3. Predictive Factors for TD in Different MDD Onset Ages With Abnormal FBG

**Figure 4:**
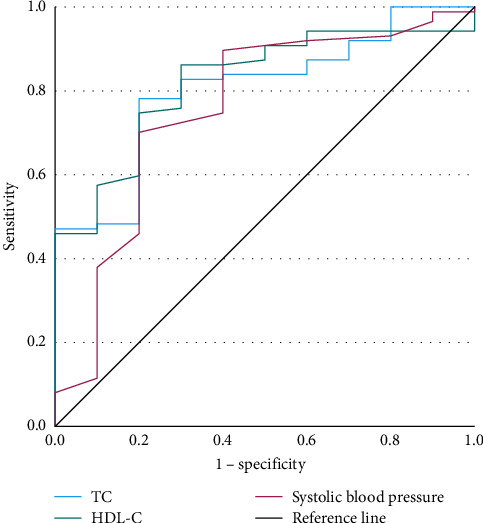
ROC curve in early-onset group with abnormal FBG. FBG, fasting blood glucose; ROC, receiver operating characteristic.

Among early-onset MDD patients with FBG abnormalities, we conducted binary logistic regressions with TD as the dependent variable (Table 6), using actual age, gender, onset age, education level, marital status, and FBG levels as control variables, and employed hierarchical regression for the analysis. We plotted the ROC curves (Figure 4) and listed parameters in Table 7.

**Figure 5:**
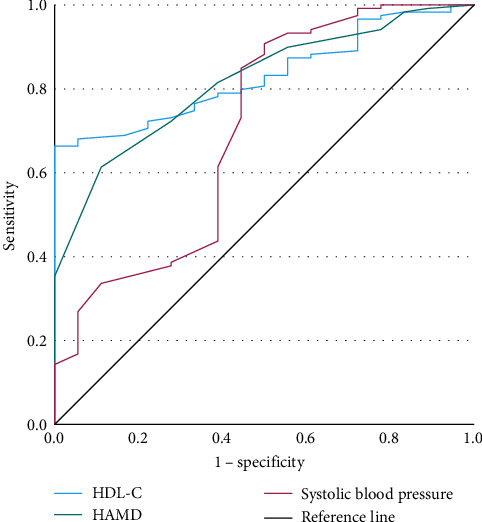
ROC curve in late-onset group with abnormal FBG. FBG, fasting blood glucose; ROC, receiver operating characteristic.

We conducted binary logistic regressions with TD of late-onset MDD patients with abnormal FBG as the dependent variable (Table 8) and plotted the ROC curves (Figure 5). The parameters of ROC curves were shown in Table 9.

## 4. Discussion

Based on our best understanding of previous literature, this study was the first to investigate TD's prevalence and pathogenic factors among FEDN MDD patients with abnormal FBG at early- and late-onset age. FEDN MDD patients were selected to exclude drug interferences on biochemical parameters, including blood glucose. The primary results of our study are threefold: (1) a significant difference in TD prevalence for MDD patients between these patients with and without abnormal FBG but not exhibited between early- or late-onset ages; (2) no significant differences of biochemical indicators were found between early- and late-onset MDD patients comorbid FBG abnormalities, and late-onset group suffered a longer duration of depression; and (3) although there were no differences, early- and late-onset MDD participants had different risk factors as well as predictive factors for TD, exhibiting more dramatic predictive factors in late-onset MDD patients.

MDD patients accompanied by FBG abnormalities have significantly higher TD prevalence and higher serum TSH levels. Compared to previous research, which investigated diabetes patients or other subjects, the prevalence of TD in our study was higher [24, 31]. This difference may be accounted by geographical location and clinical research subjects. MDD patients often exhibit impaired cerebral energy metabolism [18, 32], with higher rates of metabolic disorders [8], and MDD may further disrupt metabolism and neurotransmitters [14], leading to a higher incidence of TD. Except for MDD, patients in our study also had glucose abnormalities, exhibiting abnormal FBG. Unable to maintain normal blood glucose meant disturbed glucose homeostasis, impaired insulin secretory response, or insulin sensitivity disturbance [31], suggesting peripheral metabolism issues with thyroid hormone [33].

In this study, regardless of abnormal glucose metabolism, the prevalence of significant TDs had no differences between early-onset MDD patients and late-onset ones, which was consistent with previous findings [24, 34], though aging was with increased TD risk [35]. Early-onset MDD patients, accompanied by FBG abnormalities, had milder depressive symptoms compared to late-onset patients. This result is consistent with Charlton's work [13]. Despite differing criteria for early and late onset of MDD (age range), some studies have found similar results. Patients with adolescence MDD onset showed less impairment and psychosocial dysfunction in adulthood [34]. The adult MDD-onset patients had smaller subgenual anterior cingulate cortex volume, suggesting greater severity of depression [11]. However, although the differences in biological indicators and clinical symptoms were mainly exhibited between MDD patients with and without glucose metabolism disorder, rather than between distinct onset ages, we still found different risk factors of TD. More severe levels of depression, anxiety severity, and psychiatric symptoms may be prone to TD more likely in the late-onset group. The impaired cerebral energy metabolism in elderly MDD patients who were with glucose metabolism abnormalities caused neurodevelopmental changes and alterations in the hypothalamic–pituitary–adrenal axis (HPA) [14], resulting in disturbed dopamine and dysfunction of hypothalamus–pituitary–thyroid (HPT) axis activities [11, 18]. At the same time, aging may also lead to a decreased glucose metabolism efficiency and inability to provide sufficient energy for brain activities, affecting the normal functions of the hypothalamus and pituitary.

Different risk factors affect TD in MDD patients with glucose metabolism abnormalities depending on onset ages, and different predictive factors could detect TD in distinct onset ages. TC, HDL-C, and systolic blood pressure were primary risk indicators of TD for early-onset MDD patients with FBG abnormalities. MDD severity, HDL-C, HAMD, and systolic blood pressure were critical for TD in late-onset patients. Since the participants with abnormal FBG were not diagnosed with diabetes in this study, in addition to the depressed symptoms mentioned earlier, insulin resistance may be our initial suspected contributing factor to TD. According to further analysis, among MDD patients with abnormal FBG, 40.60% (95/234) subjects had abnormal HDL-C and/or abnormal LDL-C levels. The proportion of MDD patients with TD and FBG abnormalities was 41.71% (83/199).

Moreover, abnormal FBG MDD patients accompanying TD had higher LDL-C, lower HDL-C, and higher TC levels, consistent with previous findings that hypothyroidism could result in increased LDL-C for patients with insulin resistance and promoted hepatic cholesterol synthesis [36]. Previous research suggests that insulin resistance may occur in the peripheral tissues of the hypothyroid, leading to a decreased glucose conversion to glycogen and a decreased rate of glycolysis [37]. Additionally, the difference in FT3/FT4 value between early-onset MDD patients and late-onset MDD patients was not found in this research. Among MDD patients with abnormal FBG and TD, 67.34% were found to be obese. In the early-onset group, 68.67% of patients with both TD and FBG abnormalities were obese, compared to the ratio in the late-onset group was 67.24%. Moreover, there were no significant differences in BMI between the early-onset group and the late one, which may further indicate the role of insulin resistance in TD.

Furthermore, compared to the early-onset MDD patients with glucose metabolism abnormalities, the same predictive factors have a better predictive effect on the late-onset group. Early-onset MDD patients may be accompanied by other clinically severe manifestations, including increased suicidal thoughts, higher probability of substance dependence, and increased prevalence of sleep disorders [12, 13, 38]. Insulin resistance has been considered to be a potential associated factor for suicidal attempts in MDD, and it may be a more precise indicator to diagnose depressive symptoms compared to DSM-based diagnoses [39, 40]. These severe manifestations may affect blood glucose metabolism, thyroid function, and lipid metabolism. Furthermore, Ertekin et al. [41] found an adipokine called vaspin, which may be a predictive factor for metabolic disorders in bipolar disorder, and depressive symptoms were positively correlated with plasma vaspin levels. FBG abnormalities contribute to dyslipidemia in adolescents and older persons and subsequently affect thyroid function through LDL-C, HDL-C, and visceral fat, ultimately leading to depressive disorders symptoms [42, 43]. For late-onset MDD patients, the association between lipid metabolism abnormalities and metabolism abnormalities, depression, and metabolism abnormalities may strengthen over time, affecting thyroid function in these processes, which is consistent with our findings.

The two major strengths of our study are as follows: (1) we reveal potential internal mutual mechanisms of TD among different onset ages of MDD patients with abnormal FBG. Several studies have suggested that TD may lead to glucose metabolism disorders. Our study indicated a mutual mechanism that glucose metabolism abnormalities may also contribute to TD in MDD patients, suggesting that TD and blood glucose abnormalities in MDD patients may not only have a one-way effect. (2) Our study fills the gap in thyroid function research among patients with blood glucose abnormalities in recent years and considers the condition of MDD.

Our study has several limitations: Firstly, participants were from a single region, and these patients were gathered over several years ago. Hence, our research findings may need to be more generalizable to other regions and be necessary to gather additional individuals. Secondly, we did not collect serum levels of fasting insulin, and the participants were hard to track. Therefore, we need further study on insulin resistance and lipid metabolism abnormalities to validate our findings. Moreover, we have not gathered pertinent data regarding the patients' diet, activity, family history of thyroid genetic disorders, or metabolism because of the tracking challenges. More data in this area must be gathered for future studies. Thirdly, we did not classify the diseases caused by abnormal glucose metabolism and might have missed some meaningful findings.

## 5. Conclusion

In conclusion, MDD patients accompanied by FBG abnormalities have a higher prevalence of TD and no significant difference between early-onset and late-onset ages. For MDD patients accompanying abnormal FBG, insulin resistance and abnormal lipid metabolism based on FBG abnormalities may remarkably affect TD prevalence, which may impact late-onset MDD patients more severely. The severity of depression is the specific indicator contributing to TD for late-onset MDD patients with abnormal FBG. Considering the different pathogenic factors of TD for early- and late-onset MDD patients, patients with different onset ages must monitor different physiological parameters. Further research should focus on the pathogenic indicators of TD in MDD patients with different types of diabetes and consider more impacting factors at the same time.

## Figures and Tables

**Table 1:** Main features of participants (*N* = 1718).

Demographic variables	MDD onset age		*t*/χ^2^	*p*	Cohen's *d*/Cramer's *V*
	Less than 30 (include 30) (*n* = 749)	More than 30 years (*n* = 969)			
Course of MDD (month)	4.866 ± 3.347	7.424 ± 5.312	−12.183	<0.001	4.5608
Gender (%)	—	—	11.905 (df = 1)	<0.001	0.083
Male	290 (38.7)	298 (30.8)	—	—	—
Female	459 (61.3)	671 (69.2)	—	—	—
TD (%)	—	—	2.591 (df = 1)	0.107	—
With	439 (58.6)	605 (62.4)	—	—	—
Without	310 (41.4)	364 (37.6)	—	—	—
Educational background (%)	—	—	281.872 (df = 3)	<0.001	0.405
Middle school	42 (5.6)	371 (38.3)	—	—	—
High school	362 (48.3)	398 (41.1)	—	—	—
College	292 (39.0)	157 (16.2)	—	—	—
Postgraduate	53 (7.1)	43 (4.4)	—	—	—
Marriage (%)	—	—	721.881 (df = 1)	<0.001	0.648
Unmarried	470 (26.6)	32 (20.0)	—	—	—
Married	279 (73.4)	937 (80.0)	—	—	—
Psychotic symptoms (%)	—	—	1.134 (df = 1)	0.287	—
With	68 (9.1)	103 (10.6)	—	—	—
Without	681 (90.9)	866 (89.4)	—	—	—
LDL-C abnormalities (%)	—	—	—	—	—
With	193 (25.8)	300 (31.0)	5.566 (df = 1)	0.018	0.057
Without	556 (74.2)	126 (69.0)	—	—	—

Abbreviations: LDL-C, low-density lipoprotein cholesterol; MDD, major depressive disorder; TD, thyroid dysfunction.

**Table 2:** The prevalences of various thyroid dysfunctions (*N* = 1718).

Dysfunctions	Total MDD patients (*N* = 1718) (%)	With abnormal FBG (*n* = 234) (%)	Without abnormal FBG (*n* = 1484) (%)	*χ* ^2^	*p*	Cramer's *V*
Thyroid dysfunction	66.76	88.03	63.41	55.234	<0.001	0.197
Subclinical hypothyroidism	60.59	85.04	56.74	67.816	<0.001	0.199
A-TG abnormality	17.29	24.36	16.17	9.474	0.002	0.074
A-TPO abnormality	25.49	35.90	23.85	15.433	<0.001	0.095
Overall thyroid autoimmunity	29.28	40.17	27.56	15.523	<0.001	0.095

Abbreviations: A-TG, antithyroglobulin; A-TPO, antithyroid peroxidase; FBG, fasting blood glucose; MDD, major depressive disorder.

**Table 3:** Prevalences of TD among early-onset MDD patients (*N* = 749).

Dysfunctions	Total early-onset patients (*N* = 749) (%)	With abnormal FBG (*n* = 97) (%)	Without abnormal FBG (*n* = 652) (%)	*χ* ^2^	*p*	Crammer's *V*
Thyroid dysfunction	64.75	89.69	61.04	30.362	<0.001	0.201
Subclinical hypothyroidism	58.34	85.57	54.29	33.977	<0.001	0.213
A-TG abnormality	16.82	26.80	15.34	7.934	0.005	0.103
A-TPO abnormality	25.77	38.14	23.93	8.924	0.003	0.109
Overall thyroid autoimmunity	33.55	41.24	27.30	7.948	0.005	0.103

Abbreviations: A-TG, antithyroglobulin; A-TPO, antithyroid peroxidase; FBG, fasting blood glucose; MDD, major depressive disorder; TD, thyroid dysfunction.

**Table 4:** The prevalences of TD in late-onset MDD patients (*N* = 969).

Dysfunctions	Total late-onset patients (*N* = 969) (%)	With abnormal FBG (*n* = 137) (%)	Without abnormal FBG (*n* = 832) (%)	*χ* ^2^	*p*	Crammer's *V*
Thyroid dysfunction	68.32	86.86	65.26	25.349	<0.001	0.162
Subclinical hypothyroidism	62.33	83.94	58.77	33.914	<0.001	0.187
A-TG abnormality	17.65	22.63	16.83	2.724	0.099	0.053
A-TPO abnormality	25.28	34.31	23.80	6.876	0.009	0.084
Overall thyroid autoimmunity	29.41	55.67	27.76	7.692	0.006	0.089

Abbreviations: A-TG, antithyroglobulin; A-TPO, antithyroid peroxidase; FBG, fasting blood glucose; MDD, major depressive disorder; TD, thyroid dysfunction.

**Table 5:** Analysis of difference tests in comorbid abnormal FBG MDD patients (*N* = 234).

Indicators (M ± SD)	MDD onset age		*t*	*p*	Cohen's *d*
	Less than 30 (include 30) (*n* = 97)	More than 30 years (*n* = 137)			
Course of MDD (month)	5.041 ± 3.300	8.314 ± 5.502	−5.228	<0.001	4.717
HAMD	30.68 ± 3.118	31.42 ± 2.762	−1.921	0.056	2.914
HAMA	22.02 ± 3.643	22.26 ± 3.430	−0.503	0.616	3.520
PANSS positive subscale	9.95 ± 5.147	10.61 ± 5.946	−0.890	0.374	5.629
CGI	6.20 ± 0.799	6.27 ± 0.752	−0.724	0.470	0.772
TSH	7.11 ± 2.588	7.23 ± 2.854	−0.322	0.748	2.747
A-TG	91.36 ± 138.805	132.78 ± 343.061	−1.125	0.262	277.421
A-TPO	137.00 ± 257.443	125.28 ± 285.390	0.322	0.748	274.172
FT3	4.82 ± 0.646	4.85 ± 0.674	−0.404	0.687	0.662
FT4	16.79 ± 2.975	16.94 ± 2.992	−0.382	0.703	2.985
FBG	6.49 ± 0.384	6.52 ± 0.414	−0.613	0.540	0.402
TC	5.70 ± 1.184	5.83 ± 1.116	−0.873	0.383	1.145
HDL-C	1.14 ± 0.280	1.12 ± 0.320	0.711	0.478	0.304
TG	2.48 ± 1.036	2.42 ± 1.081	0.464	0.643	1.062
LDL-C	3.30 ± 0.893	3.26 ± 0.970	0.313	0.754	0.939
BMI	24.55 ± 2.343	24.47 ± 2.031	0.280	0.780	2.166
Systolic blood pressure	123.72 ± 9.655	123.92 ± 10.102	−0.150	0.881	9.920
Diastolic blood pressure	77.82 ± 7.056	79.01 ± 6.679	−1.303	0.194	6.838

Abbreviations: A-TG, antithyroglobulin; A-TPO, antithyroid peroxidase; BMI, body mass index; CGI, Clinical Global Impression; FBG, fasting blood glucose; FT3, free triiodothyronine; FT4, free thyroxine; HAMA, Hamilton Anxiety Rating Scale; HAMD, Hamilton Depression Rating Scale; HDL-C, high-density lipoprotein cholesterol; LDL-C, low-density lipoprotein cholesterol; MDD, major depressive disorder; PANSS, Positive and Negative Syndrome Scale; TC, total cholesterol; TD, thyroid dysfunction; TG, triglycerides; TSH, thyroid-stimulating hormone.

**Table 6:** Predictive factors for early-onset group (*N* = 97).

Variables	β	OR	95% CI	*p*	*R* ^2^
(1) TC	1.110	3.036	1.489–6.190	0.002	0.118
(2) TC	1.110	3.033	1.340–6.867	0.008	0.168
Systolic blood pressure	0.101	1.106	1.013–1.208	0.025	—
(3) TC	0.896	2.451	1.041–5.769	0.040	0.202
HDL-C	−3.238	0.039	0.001–1.071	0.055	—
Systolic blood pressure	0.090	1.094	1.003–1.193	0.043	—

Abbreviations: HDL-C, high-density lipoprotein cholesterol; TC, total cholesterol.

**Table 7:** Parameters of predictive factors for early-onset group (*N* = 97).

Indicators	AUC	95% CI	*p*	Youden index	Cutoff value	Sensitivity	Specificity	PPV	NPV
TC	0.803	0.680–0.927	0.002	0.582	5.02	0.782	0.800	91.58%	100%
HDL-C	0.821	0.706–0.936	0.001	0.562	1.390	0.862	0.700	—	—
Systolic blood pressure	0.757	0.581–0.933	0.008	0.501	120.50	0.701	0.800	—	—

Abbreviations: AUC, area under the curve; HDL-C, high-density lipoprotein cholesterol; NPV, negative predictive value; PPV, positive predictive value; TC, total cholesterol.

**Table 8:** Risk factors for late-onset group (*N* = 137).

Variables	β	OR	95% CI	*p*	*R* ^2^
(1) HDL-C	−4.140	0.016	0.002–0.119	<0.001	0.154
(2) HAMD	0.438	1.550	1.196–2.008	<0.001	0.233
HDL-C	−3.676	0.025	0.003–0.206	<0.001	—
(3) HAMD	0.491	1.634	1.224–2.181	<0.001	0.275
HDL-C	−3.366	0.035	0.004–0.325	0.003	—
Systolic blood pressure	0.088	1.092	1.020–1.169	0.012	—
(4) HAMD	0.514	1.671	1.254–2.226	<0.001	0.299
HDL-C	−4.043	0.018	0.001–0.228	0.002	—
Systolic blood pressure	0.162	1.175	1.060–1.303	0.002	—
Diastolic blood pressure	−0.149	0.862	0.746–0.996	0.044	—
(5) HAMD	0.618	1.856	1.326–2.597	<0.001	0.322
FT4	−0.244	0.784	0.621–0.990	0.041	—
HDL-C	−4.727	0.009	0.000493–0.159	0.001	—
Systolic blood pressure	0.181	1.198	1.066–1.347	0.002	—
Diastolic blood pressure	−0.182	0.834	0.710–0.9797	0.027	—

Abbreviations: FT4, free thyroxine; HAMD, Hamilton Depression Rating Scale; HDL-C, high-density lipoprotein cholesterol.

**Table 9:** Parameters of predictive factors for late-onset group (*N* = 137).

Indicators	AUC	95% CI	*p*	Youden index	Cutoff value	Sensitivity	Specificity	PPV (%)	NPV (%)
HDL-C	0.831	0.758–0.904	<0.001	0.664	1.185	0.664	1.000	87.97	50.00
HAMD	0.817	0.729–0.905	<0.001	0.502	31.50	0.613	0.889	88.64	60.00
Systolic blood pressure	0.703	0.556–0.850	0.006	0.408	114.50	0.908	0.500	88.15	100

Abbreviations: AUC, area under the curve; HAMD, Hamilton Depression Rating Scale; HDL-C, high-density lipoprotein cholesterol; NPV, negative predictive value; PPV, positive predictive value.

## Data Availability

The data that support the findings of this study are available from the corresponding author upon reasonable request. Requests may be sent to the corresponding author: zhangxy@psych.ac.cn.

## Nomenclature

MDD: Major depressive disorder
TD: Thyroid dysfunction
HAMD: Hamilton Depression Rating Scale
HAMA: Hamilton Anxiety Rating Scale
PANSS: Positive and Negative Syndrome Scale
A-TG: Antithyroglobulin
TSH: Thyroid-stimulating hormone
A-TPO: Antithyroid peroxidase
FT3: Free triiodothyronine
FT4: Free thyroxine
FBG: Fasting blood glucose
CGI: Clinical Global Impression
TC: Total serum cholesterol
HDL-C: High-density lipoprotein cholesterol
TG: Triglycerides
LDL-C: Low-density lipoprotein cholesterol
BMI: Body mass index
AUC: Area under the curve.
